# Development and Validation of a Multidimensional Expectation Questionnaire for Thalassaemia Major Patients

**DOI:** 10.5539/gjhs.v8n2p77

**Published:** 2015-06-05

**Authors:** Ioannis G. Koutelekos, Helen Kyritsi, Alexandros Makis, Constantine M Vassalos, Eftychios Ktenas, Maria Polikandrioti, Chryssa Tzoumaka-Bakoula, Nikolaos Chaliasos

**Affiliations:** 1 Medical School, University of Ioannina, Ioannina, Greece; 2 Faculty of Nursing, Technological Educational Institute of Athens, Athens, Greece; 3 National School of Public Health, Athens, Greece; 4 Medical School, University of Athens, Athens, Greece

**Keywords:** thalassaemia major, expectations, instrument validation, multiple tasks, ability

## Abstract

Nowadays, thalassaemia major (TM) patients are surviving into mature young adulthood; however, no published instrument exists to measure the expectations’ dimensionality among older TM patients in their thirties. This study seeks to validate a novel multidimensional expectation questionnaire suitable for TM patients (MEQ-TMP) reaching their fourth decade of life. In order to establish the psychometric properties of the instrument, data analysis was carried out. The principal component analysis revealed four components (‘Supportive social network’; ‘Raising one’s own family’; ‘Career advancement’; ‘Ability of daily activities’). Their cumulative contribution rate was 66.32%. Cronbach’s alpha for the total scale was 0.87. Each subscale had an alpha value above 0.70; three subscales were in the 0.80 range. MEQ-TMP reliability was proved to be good. The known-group method served as a strategy in examining the operationalisation of the questionnaire’s constructs. The present MEQ-TMP, developed for the aged group of TM patients, would be a useful tool for clinical personnel providing care to TM patients in understanding their outlook on life as they are growing up, to have better psychosocial adjustment to illness chronicity, live life as normally as possible, and fulfill their ambitions; thus enhancing their life satisfaction and quality of life.

## 1. Introduction

Beta-thalassaemia major (TM) is a recessively inherited haematological disorder. In developed countries, it is now considered a rare anaemia due to concerted prevention efforts (Vichinsky & Neufeld, 2010). In Greece, a developed country, there are still 3,241 thalassaemics, whose overall survival probability reaches 68% at the age of 40 (C. Kattamis, Sofokleous, Ladis, & A. Kattamis, 2013; Ladis, Chouliaras, Berdousi, Kanavakis, & Kattamis, 2005). After the introduction of iron chelation therapy in the 1970s, TM has shifted from being a childhood life-limiting illness into a chronic condition (Borgna-Pignatti et al., 2005; Ladis et al., 2005). Due to substantial therapeutic advances more TM patients are presumed to pass through their twenties and eventually enter their thirties (Kattamis et al., 2013; Ladis et al., 2005).

TM patients, regardless of age, are still treated mostly in children’s hospitals (for logistic reasons) and granted economic and social benefits (Kattamis et al., 2013; Compagno, 2005; Politis, 1998). However, young adults in their thirties are neither dependent as children and adolescents nor relatively independent from social roles as emerging adults in their twenties (Arnett, 2015; Benson & Furstenberg Jr., 2007). Adults between the ages of 31-40 accept their own responsibilities (Arnett, 2015; Levinson, 1986). Yet there has been no research undertaken as to what patients now living with TM in their thirties may expect for their lives in dealing with the enduring responsibilities in adulthood.

Earlier studies showed that thalassaemics may suffer from psychosocial problems and could not be integrated into normal life (Ratip & Modell, 1996; Woo, Giardina, & Hilgartner, 1985). Contradictions among different research studies have not been resolved; however, TM patients have been suggested to develop satisfactory coping mechanisms (Mednick et al., 2010; Vardaki, Philalithis, & Vlachonikolis, 2004; Bush, Mandel, & Giardina, 1996). To date, there have been only a few attempts to measure the future expectations of TM patients in the second and third decades of life (Vardaki et al., 2004; Bush et al., 1998; Di Palma, Vullo, Zani, & Facchini, 1998; Zani, Di Palma, & Vullo, 1995). The dimensions of patient life expectations have yet to be fully recognised (McCabe & Barnett, 2000; Nurmi, 1991). The present study aimed at validating a newly developed scale to measure the expectations of TM patients in their fourth decade of life.

## 2. Method

### 2.1 Development

The multi-dimensional expectation questionnaire for TM patients (MEQ-TMP), a newly designed instrument, was self-completed utilising a closed-question format. The MEQ-TMP, offered in paper-and-pencil, fully met the requirements of the current study and consisted of questions relating to TM patients’ expectations about their future. A conclusive appendix was dedicated to demographic variables.

To make a list of hypothetical domains for use in the MEQ-TMP, the researchers carried out a review of existing literature. They initially selected three very broad constructs inspired, inter alia, by a health survey conducted on TM patients (Vardaki et al., 2004), a research on their coping mechanisms (Bush et al. 1998), and earlier studies on their psychosocial aspects (Di Palma et al., 1998; Zani et al., 1995). The constructs referred to TM patients’ expectations about their 1) social, 2) family, and 3) work functioning. For each of them, 13 to 15 items were generated. Subsequent refinement was done on the basis of what was learned from 10 TM patients, 31-40 years of age, when they were asked informal interview questions, with each pilot interview lasting 20 to 30 minutes. An aspect of physical functioning was added. Four domains were then proposed reflecting expectations of TM patients in their thirties on (1) supportive social network, (2) raising one’s own family, (3) ability to perform activities of daily living (ADL), and (4) career advancement. Based on these domains, a pool of 20 potential items was finally developed. On these items, ratings were made on a four point Likert-type scale from 1=‘not at all’ to 4=‘very much’.

### 2.2 Content and Face Validity

The relevance of the questionnaire content was ascertained by a five-member, multi-disciplinary panel of health professionals. The expert judges had prolonged experience of working with TM patients over 30. Each of them was asked to review the 20 potential questions and validate whether they were representative of life expectations specific to TM patients belonging to the age group of 31-40 years, or if items would be added, modified or removed.

To ensure face validity of the questionnaire, cognitive interviews were conducted with 15 TM patients in the fourth decade of life randomly selected from a teaching children’s hospital-affiliated thalassaemia unit. The subjects were asked to complete the draft questionnaire and subsequently interviewed to assess whether each candidate question was clear, inoffensive, understandable, and presented in a logical order.

### 2.3 Data Collection

All of the 330 TM patients, who were aged 31-40 years −being born after 1974 when chelation therapy became a part of the overall TM management in Greece (Kattamis et al., 2013), and received regular blood transfusions through a thalassaemia expertise unit affiliated with a large children’s hospital located in Greece’s capital city, Athens, were targeted. Underlying chronic condition excluded 65 (19.70%) of potentially eligible TM patients. Eight (2.42%) TM patients did not meet the inclusion criterion stating that the respondents needed an education level corresponding to that of at least upper secondary school to understand the questionnaire. A total of 257 TM patients were found to be eligible for the study. The study sample size was computed by using a confidence level of 95% and a margin of error of 5%. Finally, 155 adult TM patients between 31 and 40 years of age were recruited to participate in the study over a 4-month period beginning in February 2014. All TM patients gave informed consent before they began participation in the study. Ethical approval to conduct the study was granted by the appropriate hospital authorities.

### 2.4 Construct Validity and Reliability

To make sure that the candidate items in the draft version of the MEQ-TMP did in fact measure the constructs they intended to represent, the instrument’s construct validity was investigated in the population under study. To explore the unknown structure of the items of the MEQ-TMP, a principal component analysis (PCA) was performed to identify the groups that formed the questionnaire’s subscales. The PCA (which is often considered a variant of factor analysis) is a variable-reduction procedure being exploratory in nature. All variables included in the PCA were treated as metric. One-sample Kolmogorov-Smirnov test of normality was performed (p= 0.608).

Before extracting components, the researchers examined whether the respondents’ data was worth reducing through (1) Kaiser-Meyer-Olkin (KMO) measure of sampling adequacy and (2) Bartlett’s test of sphericity. KMO values exceeding 0.70 were considered indicative of an adequate correlation among the items of the MEQ-TMP, and thus applying the PCA in this study would be useful. Bartlett’s test should reject the null hypothesis at p< 0.001 that the correlation matrix is an identity matrix to indicate that the variables were correlated, and therefore suitable for structure detection.

To estimate the number of components to be extracted, the Kaiser’s criterion was utilised based on the number of eigenvalues over 1.00 and the Cattell’s scree plot of the individual components’ eigenvalues was reviewed. Regarding our sample size, it was best to have loadings greater than 0.50 (Stevens, 2002). The cumulative proportion of total variance was also retrieved. For simplifying component interpretation, the varimax rotation was chosen as the most commonly used orthogonal rotation technique.

To test whether consistent results were yielded across items within the instrument, Cronbach’s alpha coefficient was computed for the entire scale as well as each of its subscales. For a newly developed instrument such as the MEQ-TMP, an alpha of 0.70 was considered to be acceptable (DeVon et al., 2007). To test for redundant items, the effect of removing individual questions from the scale on Cronbach’s alpha was calculated. To assess the degree of agreement among the independent raters, an intra-class correlation coefficient (ICC) value of 0.9 indicated excellent repeatability (Shrout & Fleiss, 1979).

To furher examine the MEQ-TMP reliability, every participant had the chance to complete the questionnaire again after a two-week interval. A random set of 41/109 (37.61%) TM patients, 19 (46.34%) males and 22 (53.66%) females), who were aged 34.68±4.22 years (age range of 31-40 years), accepted to answer the MEQ-TMP again two weeks later. Spearman’s rank-order correlation coefficients were used as indexes of test-retest reliability for time.

### 2.5 Item Analysis and Known Group Validity

To evaluate score distributions, mean scores, standard deviations, and ranges were calculated. For each subscale, the percentages of scores given by the respondents were also recorded. Ceilings and floor effects were considered present when more than 15% of the raters reported the best or worst possible score (Terwee et al., 2007). The assessment of the questionnaire’s ability to distinguish between subgroups of TM patients, who differed on their demographic characteristics, was based on the known subgroups demonstrating different mean scores on the MEQ-TMP using unpaired *t*-test (Hattie & Cooksey, 1984).

### 2.6 Statistical Analysis

All statistical analyses were performed using IBM® SPSS® Statistics Version 21 (IBM, Armonk, NY, USA). To assess the psychometric properties of the MEQ-TMP, several methods were applied. The statistical significance threshold was defined as p< 0.050.

## 3. Results

### 3.1 Content and Face Validity

The expert panel reached a consensus that 17 of the 20 potential items were appropriate indicators of the constructs. Regarding the assumed domain of raising one’s own family, the question Q10 (‘I expect that I will have a partner’) was removed because it was felt that it undermined questionnaire’s cohesiveness. Likewise, concerning the hypothesised construct of career advancement, the questions Q16 (‘I expect that I will choose a career based on my skills’) and Q17 (‘I expect that I will choose a career based on my family’s aspirations’) were skipped because they were thought to be superfluous. The experts considered that shortening the questionnaire without losing important data would improve TM patients’ motivation to fill out the questionnaire. To prevent careless responding, positively-worded items were also suggested. The mean age of the interviewed TM patients was 34.07±6.10 years (age range of 31-40 years), and 7/15 (46.67%) were male. The typical time required to answer all 17 candidate questions was 10 to 15 minutes. They found the 17 items easy to understand and had no difficulty answering the draft MEQ-TMP as a whole. However, taking into account the interviewees’ suggestions, some slight rewording of items was done to improve clarity.

### 3.2 Statistics and Data Analysis

The 109/155 (70.32%) consecutive, eligible patients having TM fully completed the questionnaire. Of these, 52 (47.71%) were men and 57 (52.29%) women. Their average age was 35.60±2.67 years (range 31 to 40). Table 1 demonstrates in detail their demographic characteristcs.

**Table 1:** Demographic characteristics of the respondents to the multidimensional expectations questionnaire for thalassaemia major patients

Socio-demographic characteristics	Content	Cases	Percentage (%)
Sex	Female	57	52.29
	Male	52	47.71
Educational level	High (University or above)	68	62.39
	Low (Upper secondary school)	41	37.61
Marital status	Marriage or cohabitation	40	36.69
	Singlehood	69	63.31
Employment status	Paid employment	84	77.06
	Unemployment	25	22.94

### 3.3 Construct Validity and Reliability

On the first PCA, the KMO value of 0.79 and the Bartlett’s sphericity test (χ^2^=758.64, d.f.=136, p< 0.001) displayed satisfactory results confirming the factorability of the inter-correlation matrix of the 17 candidate MEQ-TMP items. The initial solution after PCA revealed five components with eigenvalues greater than 1.00. The solution was also visually supported by the scree plot analysis. A total variance of 67.56% was yielded, and this was considered reasonable (Field, 2005). Cronbach’s alpha was calculated and was 0.82 indicating a high correlation between the revised draft MEQ-TMP items. To test for redundant items, the effect of deleting individual questions on alpha was also computed. The removal of questions Q13 (‘I expect that my health condition will prevent me from doing the things that my healthy friends can do’) and Q14 (‘I expect that when doing things I will worry more about my health than most people will’) increased coefficient alpha from 0.82 to 0.84, and 0.83, respectively. It was therefore suggested that these items were redundant and should be dropped from the questionnaire. A PCA was re-run with the remaining 15 items. The KMO value of 0.81 was greater than 0.70 indicating a good correlation among the remaining MEQ-TMP items. The significant Bartlett’s test (χ^2^=706.44, d.f.=105, p< 0.001) also confirmed that this data set was suitable for implementing PCA. Based on Kaiser’s criterion, four principal components were now postulated (Table 2). A review of the scree plot compiled on the repeated PCA also suggested that a four-component model was viable. The four dimensions in the component space accounted for 66.32% of the variance. Item loading values were above the cut off value.

**Table 2:** Subscales of the multidimensional expectations questionnaire for thalassaemia major patients

Questions	Individual loadings				Communalities

	Subscales				

	1^a^	2^b^	3^c^	4^d^	
Q01, I expect that there will be several people in my life, whom I trust, to help me solve my problems	0.83	0.11	0.09	0.02	0.71
Q02, I expect that when Ifeel lonely there will be many people that Ican talk to	0.80	0.11	0.09	0.16	0.69
Q03, I expect that I will always be surrounded by friends	0.71	0.17	0.23	0.18	0.61
Q04, I expect that when I need advice there will always be someone to turn to	0.68	0.14	0.07	0.20	0.53
Q05, I expect that I will often been invited to do social activities	0.74	0.13	0.11	0.12	0.58

Q06, I expect that I will have a normal family’s life	0.38	0.72	-0.04	-0.01	0.66
Q07, I expect that I will raise my own family	0.05	0.73	0.21	0.06	0.58
Q08, I expect that when I will handle family demands as satisfactorily as everyone else my age	0.11	0.73	0.16	0.43	0.75
Q09, I expect that I will have the same chance to raise my own family aw everyone else my age	0.15	0.82	0.13	0.27	0.78

Q11, I expect that I will be able to do in daily life what everyone else my age does	0.23	0.36	0.13	0.79	0.82
Q12, I expect that I will have the physical skills to do everyday things like everyone else my age	0.31	0.13	0.12	0.82	0.79

Q15, I expect that I will be proactive at work	0.18	0.34	0.58	-0.14	0.51
Q18, I expect that I will choose the job I love	0.32	0.10	0.58	0.08	0.46
Q19, I expect that it will be easy for me to advance my career	0.05	-0.06	0.85	0.33	0.83
Q20, I expect that I will have a better chance for career advancement than most of my co-workers	0.03	0.15	0.80	0.06	0.66

Variance (%)	22.00	17.42	14.90	12.00	

a, Subscale 1 ‘Supportive social network’; b, Subscale 2 ‘Raising one’s own family’; c, Subscale 3 ‘Career advancement’; d, Subscale 4 ‘Ability of daily activities’.

*Note*. The English version of the questionnaire presented here is by no means linguistically validated.

Coefficient alpha for the 15-item scale was computed and was 0.87 far exceeding the minimum value for a new tool. The researchers stopped the item elimination process because reliability coefficient was found to decrease if any of the items were deleted. Each of the four subscales had an acceptable alpha greater than 0.70. No item proved to be unreliable (Table 3). The ICC was 0.87 (almost perfect) and corresponding 95% confidence interval 0.83-0.90 (Sharrack, Hughes, Soudain, & Dunn, 1990). A sample of 25/41 (60.98%) TM patients, 11 (44.00%) males and 14 (56.00%) females, who were 31 to 40 years old (mean age, 33.68±4.80 years), fully completed the MEQ-TMP in two test sessions. In that particular sample, the MEQ-TMP had good test-retest reliability; correlation coefficients were in the range 0.51 to 0.96 that were statistically significant, with the respecrive p-values being below 0.001 (Taylor, 1990).

**Table 3:** Assessment of internal constistency for each subscale of the multidimensional expectations questionnaire for thalassaemia major patients using Cronbach’s alpha

Subscales	Items	Cronbach’s alpha	Cronbach’s alpha, if item deleted
1, ‘Supportive social network’	No of items: 5 (Q1−Q5)	0.85	
	Q1		0.80
	Q2		0.80
	Q3		0.82
	Q4		0.84
	Q5		0.83

2, ‘Raising one’s own family’	No of items: 4 (Q6−Q9)	0.81	
	Q6		0.80
	Q7		0.80
	Q8		0.75
	Q9		0.71

3, ‘Career advancement’	No of items: 4 (Q15, Q18−Q20)	0.73	
	Q15		0.71
	Q18		0.71
	Q19		0.58
	Q20		0.65

4, ‘Ability of daily activities’	No of items: 2 (Q11, Q12)	0.80*	

* , Spearman-Brown reliability estimate

*Note*. Spearman-Brown coefficient was considered to be more appropriate to measure the reliability of the two-item subscale than Cronbach’s alpha (Eisinga, te Grotenhuis, & Pelzer, 2013).

### 3.4 The Final MEQ-TMP

The final MEQ-TMP included four subscales named after examination of the item content loading on each component (Table 2). The subscale 1 was named ‘Supportive social network’ after the item Q01 with the highest loading (0.83). This subscale indicated the TM patients’ expectation of having a social network, consisted of five items, and explained most (22.00%) of the total variance. The subscale 2, named ‘Raising one’s own family’ after the item Q09 of high loading (0.82), expressed the TM patients’ expectation of having a family, included four items, and interpreted 17.42% of the variance. The subscale 3 was named ‘Career advancement’ after its most weighted (0.85) item that is Q19. This subscale referred to the TM patients’ expectation of developing a career, included four items, and accounted for 14.90% of total variance. The subscale 4, named ‘Ability of daily activities’, focused on the TM patients’ expectation of having ability to perform everyday tasks, consisted of two items (Q11, Q12) with high loadings (0.82 and 0.79, respectively), and explained 12.00% of total variance.

### 3.5 Ceiling-Floor Effect and Operationalisation

The distribution of total scores for each subscale is shown in Table 4.

**Table 4:** Respondent’s score for each subscale of the multidimensional expectations questionnaire for thalassaemia major patients

Subscales	Score					

	Mean	Standard deviation	Minimum	Maximum	Respodents at floor (%)	Respodents at ceiling (%)
1, ‘Supportive social network’	3.03	0.65	1.60	4.00	4.59	11.93
2, ‘Raising one’s own family’	2.93	0.78	1.25	4.00	2.75	14.68*
3, ‘Career advancement’	2.76	0.77	1.00	4.00	2.75	11.01
4, ‘Ability of daily activities’	3.20	0.72	1.00	4.00	0.92	28.44

The subscale 4 had the greatest ceiling effect (28.44%) of the MEQ-TMP subscales. No floor effect was detected.The results on the relationship of the MEQ-TMP constructs with the TM patients’ demographic variables are summarised in Table 5. Comments on results are ignored unless statistically significant differences were yielded.

**Table 5:** Mean scores of the subscales of the multidimensional expectations questionnaire for thalassaemia major patients by demographic characteristics

	Sex			Education level		

	Female	Male		High	Low	
			
Subscales	Mean score	Mean score	p	Mean score	Mean score	p
1, ‘Supportive social network’	3.05	3.00	0.719	3.04	3.00	0.780
2, ‘Raising one’s own family’	2.98	2.88	0.501	3.00	2.82	0.230
3, ‘Career advancement’	2.93	2.58	0.019*	2.90	2.54	0.017*
4, ‘Ability of daily activities’	3.31	3.08	0.100	3.36	2.93	0.004*

* , statistically significant (To be continued)

**Table 5:** (cont.) Mean scores of the subscales of the multidimensional expectations questionnaire for thalassaemia major patients by demographic characteristics

	Marital status			Emploment status		

	Marriage or cohabitation	Singlehood		Paid employment	Unemployment	
			
Subscales	Mean score	Mean score	p	Mean score	Mean score	p
1, ‘Supportive social network’	3.22	2.92	0.012*	3.07	2.87	0.176
2, ‘Raising one’s own family’	2.92	2.95	0.825	3.10	2.39	0.001*
3, ‘Career advancement’	2.86	2.70	0.297	2.79	2.68	0.547
4, ‘Ability of daily activities’	3.30	3.14	0.259	3.23	3.10	0.444

* , statistically significant

## 4. Discussion

The MEQ-TMP was developed and validated as a new instrument to accurately assess expectations among adult TM patients in their thirties. To our knowledge, this is the first questionnaire measuring expectations about the future as a multidimensional concept while solely exploring the perspective of TM patients aged 31-40 years in line with their developmental stage (Levinson, 1986; Vaillant & Milofsky, 1980). Future anticipation differs among different age groups (Lachman, Röcke, Rosnick, & Ryff, 2008). There were a few single- bi- and multi-dimensional instruments, almost particularly for thalassaemics in adolescence or early adulthood, which measure life expectations (Vardaki et al., 2004; Bush et al., 1998; Di Palma et al., 1998; Zani et al., 1995). Regarding adolescent TM patient life expectations, the Ferrara study group from Italy (Di Palma et al., 1998; Zani et al., 1995) created ad hoc a non-validated questionnaire covering multiple needs related to their age (family relationships, level of social integration, heterosexual relationship, self concept, and coping strategies), while a U.S. study (Bush, 1998; Bush et al., 1998) carried out exploratoty factor analysis on responses to a 13-item questionnaire resulting in a single factor solution for their outlook on life. Concerning young adult TM patients, the Italian research team developed ad hoc a non-validated scale measuring their perspectives on marital life alone (DiPalma et al., 1998). In a Greek study (Vardaki et al., 2004), principal component analysis was performed on six items ranked by young adult TM patients in order of importance yielding a two-factor solution corresponding to long-term and realistic expectations from their life.

Commonly thought of as a starting point for questionnaire design, preliminary hypotheses were first generated on the basis of a systematic search of the literature. These constructs were further refined through pilot interviews. The researchers took into consideration the education level of the respondents and the clarity of the potential items so that the latter could be used for the MEQ-TMP development.

After a pool of potential items was generated, an independent panel of experts was used to assess the questionnaire’s content validity, thus ensuring that the domains explored were perceived as relevant to expectation concept among TM patients in the fourth decade of life. Despite being the lowest form of validity, face validity provided important information on the process of defining variables into measurable factors. TM patients belonging to the same age group as our study participants suggested minor changes. It was considered that the amended questionnaire could be readily administered to TM patients by self-administration. The small number of items included in the questionnaire resulted in short completion time.

As shown in Table 3, the reliabilities found for two MEQ-TMP subscales were greater than the recommended 0.80 for clinical use (Steiner, 2003). Subscales 3 and 4 fell short of 0.80, but were at least above 0.70 recommended for research purposes. The concordance of scores achieved by the respondents was high. Despite the small number of questions, test-retest reliability was proved to be good in the sample eventually studied. These raters did not alter their judgements, thus implying that the questionnaire might be more widely implemented. The two-week time interval between test and retest visits might seem to have prevented participants from remembering their responses and responding based on recall.

The sample size of the older TM patients participating in the study was in terms of the arbitrary rule of thumb suggesting that a sample size of 100 should be reasonable for inferential statistics in order to measure any of the MEQ-TMP psychometric properties (Hair, Jr., Black, Babin, & Anderson, 2009). In the study, the subjects-to-variables ratio of 6.41:1 was larger than 4:1, and thus the stability of a factor solution was ensured. (MacCallum, Widaman, Preacher, & Hong, 2001) The final PCA succeeded to add empirical weight to the four domain structure that had been hypothesised when designing the questionnaire. All items loaded above the cut off value on their respective subscale. The ideal situation would be if there were at least three items in each construct (Eisinga et al., 2013). However, only two questions (Q11, Q12) were included in the subscale 4 ‘Ability of daily activities’ that the research teams first introduced as a specific construct. Adequate internal reliability of the subscale was predicted (Table 3). The two items went well with each other, with an item-total correlation coefficient of 0.67; and were highly loaded on the above subscale (Table 2). ADL performance allows individuals to live independently in a community (Bookman, Harrington, Pass, & Reisner, 2007). Independent living skills are needed to manage the practical responsibilities of mature young adulthood (Arnett, 2015; Benson & Furstenberg Jr., 2007). Therefore, the subscale 4 was suggested to measure an element of life expectations of TM patients in their thirties that is different than the rest of the MEQ-TMP components. Retaining these items would enrich the questionnaire enabling us to better differentiate future perspectives of adult TM patients who have reached their fourth decade of life owing to various advances in medical treatment (Kattamis et al., 2013; Ladis et al., 2005). As demonstrated in Table 4, they scored high on expecting to be able to achieve a normal life as they moved beyond the decades-old societal stigma of physical disability (Zani et al., 1995).

The MEQ-TMP seems to demonstrate different scores for TM patients’ subgroups known to vary on their demographic variables, and thus it would be useful in monitoring the TM patients during their course of life. Intimate relationships allow people to form a strong social network (Miller & Perlman, 2008). The statistically significant finding that TM patients, who were married or living with a partner, expected to be socially supported is congruent to this. An essential part of the identity of young adults in their thirties involves taking full responsibilities for their lives (Levinson, 1986). Responding to age-related realities, young adults are striving to consolidate their goals (Vaillant & Milofsky, 1980). Expectations tend to be task-oriented (Seginer, 2008). Our 31 to 40 year olds expected to accomplish tasks that are normative for their age such as those of entering the marriage market to start a family and having career ambition (Arnett, 2015). In keeping with the notion that active parenting is related to parents’ undertaking paid work (Repetti & Wang, 2014), the TM patients in their thirties anticipated more strongly to raise a family when being in paid employment. In today’s Western societies, young women tend to surpass men their age in career ambition (Patten & Parker, 2012). In congruence to this, the female TM patients in their thirties were significantly more likely to look for career advancement than the male TM patients their age. Career achievement in the thirties was found to be associated with teenage job aspirations influenced, among others, by educational level (Domenico & Jones, 2006; Schoon, 2001). In line with this, the TM patients’ expectations for having successful career in their thirties were found to be statistically significantly associated with a high level of education achieved at a younger age. A higher educational attainment has also been shown to negatively influence functional limitations on daily activities (Bound, Schoenbaum, & Waidmann, 1995). In accordance with this, a highly significant association emerged between a higher educational level and the TM patients’ capacity to manage the day to day demands of everyday life while facing the challenge of independent living.

### 4.1 Limitations

The final shortened version of the MEQ-TMP was applicable to the data set provided by the study. It is of note here that the reliability and validity testing undertaken was for the complete set of items. If any new items are to be added or any existing parts of the current questionnaire are to be edited, the questionnaire should undergo further validation testing. The English version of the questionnaire presented here (Table 2) has not been validated. However, the original MEQ-TMP does not contain any items specifically related to Greek culture. To compare international data, it is possible to translate the questionnaire in a linguistically appropriate manner.

As yet, there has been no ‘gold standard’, obtained at approximately the same point in time, for independently assessing the multidimensional conceptualisation of life expectations amongst thalassaemics in their thirties. No concurrent-validity comparison could be made to investigate the relative performance of the MEQ-TMP compared with other available measures. The instrument’s development might have introduced biases. Therefore, the research team could not conclude whether or not this measure was the most appropriate in the given research setting. However, the known-group method served as a strategy in examining the operationalisation of the questionnaire’s constructs (Sharrack et al., 1990).

The subscale 4 ‘Ability of daily activities’ had an excessive ceiling effect implying that the MEQ-TMP highest score was unable to assess the TM patient’s level of ability. However, the researchers’ intention was to determine whether or not the TM patients had the ability of performing the same normal activities of daily life as their same-aged peers. Although the issue of ceiling effect observed in this study may not be considered to be a problem, a further approach based on different levels of complexity in relation to the daily activities ability of TM patients in their thirties would fix this issue.

### 4.2 Conclusion

A 15-item scale was developed to measure the construct of expectations about future life events among TM patients aged 31-40 years. The MEQ-TMP was relatively short, yet had good internal consistency and repeatability. The research team is planning to assess the questionnaire’s reliability over time in a larger population. The findings provided empirical evidence for a multi-dimensional approach to the expectations of TM patients in their thirties. More research is needed to document the relationship of the questionnaire with the psychosocial factors possibly moderating TM patients’ expectations. A further evaluation would ensure whether the MEQ-TMP is transferable across patients with other chronic diseases to monitor their expectations of achieving their goals while being adapted to their condition.

### 4.3 Relevance to Clinical Practice

Patients of different ages may have different expectations about their future. The MEQ-TMP, as developed here, may be like a potentially useful tool to enable nurses and other clinical personnel, who have been providing care to TM patients since childhood when their patients started transfusions, in understanding their outlook on life as they are growing up and accomplishing the tasks appropriate to their age. The novel tool may also provide an adequate basis for helping young adult patients, who have been living with TM for more than 30 years, to have better psychosocial adjustment to illness chronicity, live life as normally as possible, and fulfil their ambitions; thus enhancing their life satisfaction and quality of life.
